# Implementing the 3T‐approach for cervical cancer screening in Cameroon: Preliminary results on program performance

**DOI:** 10.1002/cam4.3355

**Published:** 2020-08-05

**Authors:** Juliette Levy, Marie de Preux, Bruno Kenfack, Jessica Sormani, Rosa Catarino, Eveline F. Tincho, Chloé Frund, Jovanny T. Fouogue, Pierre Vassilakos, Patrick Petignat

**Affiliations:** ^1^ Faculty of Medicine University of Geneva Geneva Switzerland; ^2^ Faculty of Medicine and Pharmaceutical Sciences University of Dschang Dschang District Hospital Dschang Cameroon; ^3^ Gynecology Division Department of Gynecology and Obstetrics University Hospitals of Geneva Geneva Switzerland; ^4^ Geneva School of Health Sciences HES‐SO University of Applied Sciences and Arts Western Switzerland Geneva Switzerland; ^5^ Faculty of Medicine and Biomedical Sciences Centre Hospitalier Universitaire (CHUY) Yaoundé Cameroon; ^6^ Department of Obstetrics and Gynecology Bafoussam Regional hospital Bafoussam Cameroon; ^7^ Geneva Foundation for Medical Education and Research Genève Switzerland

**Keywords:** cervical cancer, management, prevention, screen‐and‐treat, sub‐Saharan Africa

## Abstract

Option recommended by World Health Organization (WHO) includes human papillomavirus (HPV) primary screening followed by visual inspection with acetic acid (VIA) triage. We implemented a program based on a 3T‐approach (Test‐Triage and Treat). Our objective was to verify the effectiveness of the program by defining a set of performance indices. A sensitization campaign was performed in Dschang (Cameroon) and women aged 30‐49 years were invited to participate for screening based on the 3T‐approach. Participants performed HPV self‐sampling (Self‐HPV), analyzed with the point‐of‐care Xpert HPV assay followed by VIA/VILI triage and treatment if required. Key performance indicators (KPIs) for screening, diagnosis, treatment and follow‐up were defined, and achievable targets were described for which the approach is likely to be running optimally. A total of 840 women with a mean age of 39.4±5.9 years participated. The KPIs included (i) the screening rate (8.4% at 7 months, target =20% at 12 months), (ii) HPV positivity rate (19.8%, expected range 18‐25%), (iii) compliance to referral to VIA/VILI and complete test (100%, target >90%), (iv) compliance to referral to thermal ablation (100%, target >90%), (v) VIA/VILI positivity rate (50.6%, expected range 45‐55%), (vi) a single visit from diagnostic to treatment (79.8%, target >80%), (vii) compliance to follow‐up at 1 month (96.4%, target >80%) and (viii) at 6 months (70.6%, target >80%). Program performance based on the single‐visit 3T‐approach corresponded to defined targets and preliminary results support adequateness of KPIs for periodic monitoring.

## Graphical abstract

The study shows the performance of screening for cervical cancer in a single visit, using the 3T strategy, for Test‐Triage and Treat in low‐income countries Our results will be of great interest especially those designing and conducting women’s health screening programs in low‐income countries. Improved primary screening, diagnosis, treatment, and follow‐up, such as in the single‐visit 3T approach to screen cervical cancer in Cameroon, will substantially reduce the incidence of preventable or treatable diseases in low‐ and middle‐income countries.

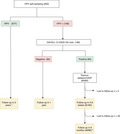

## 1 INTRODUCTION

Cervical cancer (CC) is the first cause of women cancer death in sub‐Saharan Africa.^1^ Every year, this cancer kills 266’000 women and 90% of them are coming from low and medium‐incomes countries (LMIC).^2^ This inequality is directly linked to the lack of CC prevention, early detection and treatment of precancerous lesions.

In high‐income countries having screening programs, there has been a significant decrease in CC incidence and mortality.^3^ However, this decline has not occurred in LMIC, mainly because of the absence of prevention programs, lack of infrastructure and trained caregivers. This leads to late management of invasive cancer, for which there is no more curable treatment available. In Cameroon, CC is the second most important cancer in women, with 2356 new cases detected in 2018 and 1546 related deaths.^4^ These issues highlight the need for policy makers to focus efforts on improving prevention of CC in the country.

In LMIC, the World Health Organization (WHO) recommends primary human papillomavirus (HPV) screening followed by visual inspection with acetic acid (VIA) for triage of HPV‐positive women and treatment if necessary.^4^ Screening with HPV testing provides two significant advantages. First, it can be carried out on self‐collected vaginal samples (Self‐HPV), thus obviating the need of a speculum examination and second, some platforms perform rapid HPV tests allowing for results at the point of care. The combination of these two advantages contributes to propose a screening and treatment approach in a single visit. This strategy increases program effectiveness and makes efficient use of available human and financial resources as well as to reduce loss of follow‐up.^5^, ^6^ Triage evaluation of HPV‐positive women using VIA and VILI can be enhanced by smartphone digital images taken before and after application of acetic acid (D‐VIA) and after Lugol's iodine (D‐VILI). This innovation is important for settings where the use of colposcope may be economically prohibitive.^7^

We implemented in Cameroon a “single‐visit approach,” called 3T‐approach (Test, Triage and Treat) based on Self‐HPV testing, triage with VIA/VILI coupled by digital photographs (D‐VIA/D‐VILI) and treatment with thermal ablation or Loop Electrosurgical Excision Procedure (LEEP) if needed.^5^, ^8^

The success of a screening program depends on a well‐organized patient itinerary as well as a well‐trained staff able to clearly explain the process and get women adhesion. To monitor the quality of the program and to ensure that desired outcome are achieved, it is necessary to use a data collection tool to identify problem and plan corrective actions..^9^, ^10^

The aim of this study was to assess quantitatively how a screening program based on the 3T‐approach can be evaluated and monitored. For this purpose, over a 7‐month pilot phase, we defined a set of key performance indicators (KPIs) including corresponding targets or standards against which performance was assessed.

## 2 METHODS

### 2.1 Health facility and program description

The 3T‐approach was introduced in September 2018, in the District Hospital of Dschang, a rural city located in the West Province of Cameroon, with an estimated population of 220’000 inhabitants. The program, which is scheduled for 5 years, is based on the WHO recommendations to screen women aged 30‐49 years at least once in a 5‐year period. According to the national census, we estimated that about 10’000 women should be screened in order to obtain an 80% coverage of this targeted population.

Regarding the recruitment, at the beginning of the campaign, women living in town were brought in by word of mouth. Then we used different methods such as talks in community centers, churches and women association's group. We have made radio advertisements and banners. And then we mobilized and trained community health workers to reach out to women living in different rural area to improve women's participation.

The local team organized a one‐hour session daily for the women willing to be screened, in order to sensitize participants about CC, HPV and CC prevention. After this information session, all women aged 30‐49 years were included in the study after full understanding of the procedure, and an informed consent form, available in French and in English, was signed. Exclusion criteria were pregnancy and previous total hysterectomy. This study was approved by the Ethical Cantonal Board of Geneva, Switzerland and the Cameroonian National Ethics Committee for Human Health Research. It was registered at ClinicalTrials.gov number NCT03757299.

The study procedure was divided in four steps: (a) a case report form (CRF) on socio‐demographic data and medical history was completed by a midwife; (b) woman performed a Self‐HPV with a flocked swab (Self Collection FLOQSwab™, Copan), and the result was given within 1 hour. During this time, the women completed a questionnaire on the acceptability of Self‐HPV; (c) if HPV was negative, women were reassured and advised to repeat the test in 5 years; if HPV was positive, a pelvic examination was completed by the midwife. VIA, D‐VILI; (d) if the provider visualized an acetowhite or iodo‐negative lesion, the procedure was completed by thermal ablation (WISAP®; Medical Technology GmbH, Brunnthal/Hofolding, Germany) or the patient was referred for LEEP if non eligible. In case of invasive cancer, the woman was led to the gynecologic department to get a full assessment and treatment that was completely covered by the program. All data regarding the gynecological examination and treatment were recorded in the patient's file.

For quality control, cytology and biopsies were performed for all HPV positive women, but results are not reported here. Local staff was trained through an e‐learning platform managed by physicians from the University of Geneva and Dschang. One week of theoretical course followed by one week of practical training were required. After two weeks of training, review of normal and abnormal cervical photographs as well as clinical sessions to observe and practice the technique were organized. At the end of the training, a theoretical and practical exam was performed to ensure that all the necessary skills were acquired. Health care providers were certified by the University of Geneva and Dschang. After the implementation of the 3T‐approach physicians and consultants continued to provide training to nurses, to verify quality improvement efforts and help manage difficult cases. The files of digital smartphone photographs are used for review and quality improvement.

### 2.2 Self‐vaginal sampling for HPV testing/ GeneXpert®

Participants performed self‐HPV by themselves and those having difficulties were assisted by the health staff. The swab was then plunged into a vial containing 20 ml NaCl 0.9% solution, and then vortexed for 30‐45 seconds. One milliliter of the solution was collected with a Pipetman and then transferred into a single‐use disposable cartridge that holds PCR reagents of the GeneXpert® machine (GeneXpert®IV. Cepheid, 2015. Sunnyvale, California, USA). The Xpert HPV assay^®^ uses PCR to detect the DNA of 14 high risks HPV. Specifically, it identifies types HPV 16 and HPV 18/45 in two distinct detection channels (channel 1 and 2), and reports 11 other high‐risk types (31, 33, 35, 39, 51, 52, 56, 58, 59, 66, and 68) in a pooled result, after detection in three distinct channels.

### 2.3 Triage with VIA/VILI

During pelvic examination, VIA and VILI inspection were done to detect precancerous lesions. Only lesions localized into the transformation zone were considered as significant.^11^ We applied a cotton‐swab soaked with acetic acid on the cervix and waited one minute to evaluate the results. We used “relaxed WHO criteria” meaning that we have considered that any aceto‐white lesion including faint whitening were considered as VIA positive.^12^ A VILI‐positive test was defined as an iodo‐negative area corresponding to an aceto‐white area.

### 2.4 Smartphone (D‐VIA/VILI)

We used a Samsung Galaxy® S5 (16 Megapixels) and the application named “Exam”.^13^, ^14^ First, a native picture was taken, then after VIA and VILI. The ExamApp classified pictures in patient file.^7^ Digital imaging of the cervical was used for peer review, quality assurance, continuing medical education and access to expert opinion if needed. Diagnosis and treatment decision were based on a combination of VIA/VILI and D‐VIA/VILI interpretation. So, the final diagnosis was made with the combination of the two methods. If we have one negative and one positive, results were considered as positive. Hereafter, VIA/VILI combined with D‐VIA/VILI were referred in the document as VIA/VILI.

### 2.5 Thermo‐ablation

We used a thermocoagulator (WISAP®; Medical Technology GmbH, Brunnthal/Hofolding, Germany), which heats a probe at 100° Celsius. This probe was applied on the precancerous lesion for 60 seconds to eliminate the abnormal zone.^15^

### 2.6 Follow‐up of positive women at one month and six months

Women who received a treatment (thermal ablation or LEEP) are reconvened one month and six months after the procedure. The control at one month consists of a clinical exam. Then at six month they will have an HPV test, a VIA/VILI inspection and cytology and biopsies. If the cytology or biopsy is positive, they will be reconvened for treatment.

### 2.7 Health information system

The sociodemographic and medical data and health outcomes were first collected by midwives on CRF‐paper during anamnesis and screening. All data including treatment and follow‐up were later registered on SecuTrial® software database by trained doctor. Data are monitored monthly. Screening results and treatment decision of midwives is recorded daily and transcribed on our database, as well as the monthly assessment of their clinical practice.

### 2.8 Key performance indicators (KPIs)

Performance threshold have been defined for each KPI with the aim of attain an achievable target or expected range that was developed according to previous studies and local conditions^5^, ^16^, ^17^, ^18^ as well from those issued by the WHO.^19^ A total of eight KPIs from screening diagnosis to treatment and follow‐up have been developed and include (a) the screening rate of the target population, (b) the percentage of HPV‐positive women, (c) the percentage of HPV‐positive women who had VIA/VILI triage, (d) the percentage of VIA/VILI positive, (e) the percentage of VIA/VILI positive women who received treatment, (f) the percentage of women having complete the single‐visit 3T‐approach, (g) the percentage of treated women having follow‐up at 1, and (h) 6 months.

### 2.9 Statistical analysis

Quantitative data were entered and analyzed using Stata Statistical Software Release 13 (StataCorp LP, College Station, TX, USA). A descriptive analysis was conducted; categorical variables were summarized with frequencies and percentage, and continuous variables were summarized with means and standard deviations (SD). Women's socio‐demographic and medical data were collected, stored, and managed by the SecuTrial online database.

## 3 RESULTS

### 3.1 Socio‐demographics

A total of 840 women participated in the CC screening campaign during the study period (Table 1). Average age of participants was 39.4 ± 5.9 years old. Majority of women (n = 729; 86.7%) were married or in relationship. Most of them (n = 696; 82.9%) had completed high school and had a professional activity (n = 699; 83.2%). The average age of menarche was 14.6 ± 1.8 years old. Mean age of first sexual intercourse was 18.1 ± 2.9 years old and a majority (n = 567, 67.5%) has had between 2 and 5 partners. The socio‐demographics are comparable to those obtained in previous studies in Cameroon.^20^

**Table 1:** Socio‐demographic and clinical characteristics of study participants

Variable	N	%
Total	840	
Age (mean ± SD), y	39.4 ± 5.9	
Age groups, y		
30‐34	216	25.7
35‐39	196	23.3
40‐44	230	27.4
>45	198	23.6
Marital status		
Single	68	8.1
Married/ In relationship	729	86.7
Divorced/Widow	43	5.1
Education		
Unschooled/ Primary education	144	17.1
Secondary education/ University	696	82.9
Age at menarche (mean ± SD), y	14.6 ± 1.8	
<13 years	111	13.2
13‐15 years	473	56.3
16‐20 years	256	30.5
Age of first intercourse (mean ± SD), y	18.1 ± 2.9	
<16 years	104	12.4
16‐18 years	441	52.5
>18 years	295	35.1
Number of sexual partners lifetime (mean ± SD)	4.2 ± 3.6	
0‐1	105	12.5
2‐5	567	67.5
6‐9	120	14.3
>10	48	5.7
Overall HPV prevalence	166	19.8
VIA/VILI done	166	19.8
VIA/VILI		
Positive	84	50.6
Negative	82	49.4
Decision to treat		
Final decision to treat	84	50.6
Coinfection HIV‐HPV		
HIV positive	10	6.0
HIV treated	9	90.0
Status HIV known^a^	163	98.2
Status HIV unknown	3	1.8

Abbreviations: N, number; SD, standard deviation; y, years; HPV, human papillomavirus; VIA, visual inspection with acid acetic; VILI, visual inspection with Lugol's iodine; HIV, human immunodeficiency virus.

^a^ Anamnestic data.

### 3.2 HPV prevalence and types

Overall, 166 patients were HPV positive, corresponding to a positivity of 19.8%. The results showed that 3.6% (n = 6) of women were infected by HPV 16, 13.9% (n = 23) by 18/45 and 77.1% (n = 128) by “other” HR HPV. Combined infections were found in 4.1% (n = 9) of women (Table 2). Of the 166 HPV positive patients, 6% (n = 10) were co‐infected with HIV and 5.4% (n = 9) were under Antiretroviral therapy (ART). VIA/VILI screening was performed on all 166 patients and 84 (50.0%) were VIA/VILI positive.

**Table 2:** Types of HPV detected in 166 infected women

Xpert typing	HPV 16 Channel 1	HPV 18/45 Channel 2	11 Other HR HPV 3 Channels^a^
Single HPV detection (N)	6 (3.6%)	23 (13.9%)	128 (77.1%)
Additional HPV detection (N)			
HPV 16	—	2 (1.2%)	2 (1.2%)
HPV 18/45	2 (1.2%)	—	5 (3%)
“Other” HR HPV	2 (1.2%)	5 (3%)	—

Abbreviations: N, number; HPV, human papillomavirus; HR, high risk.

^a^ Detection in three separate channels for 11 HR HPVs to generate a pooled result: Channel 3: HPV31, −33, −35, −52, and −58; Channel 4: HPV51 and −59; Channel 5: HPV39, −56, −66, and −68.

### 3.3 Screening, triage, and treatment adherence

**Figure 1:**
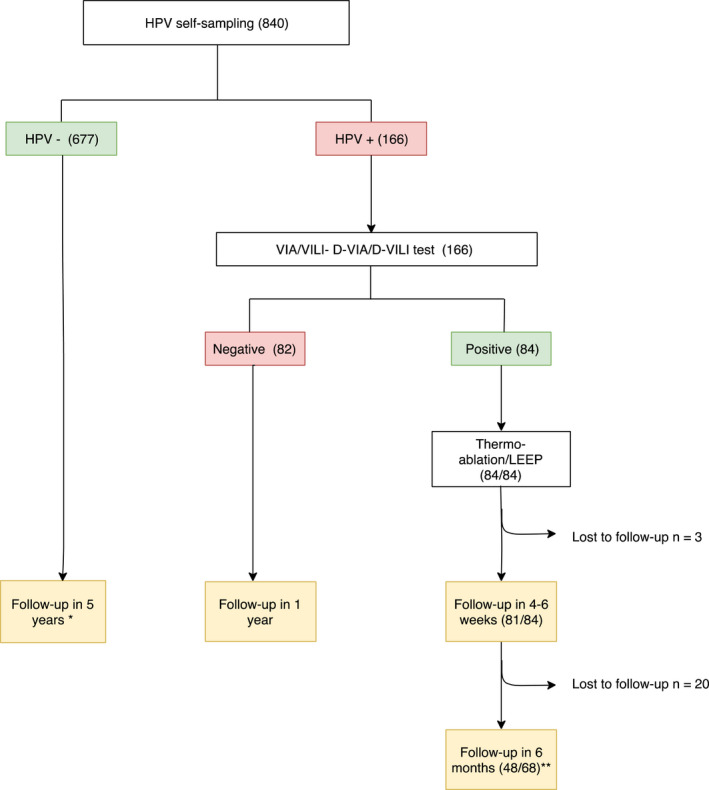
Study flowchart and results. Note: (n), number of patients, HPV, human papillomavirus; VIA, visual inspection with acid acetic; VILI, visual inspection with Lugol's iodine; D‐VIA/D‐VILI, Digital‐VIA/Digital‐VILI. *If HIV positive, follow‐up in 3 years. ** Patients included in the study who were able to benefit from their visit at 6 months

The process was accepted by all participants and performed according to protocol. The GeneXpert^®^ machine worked as expected and delivered the results in 1 hour. In addition, it was possible to repeat the test if it turned out to be uninterpretable due to insufficient sampling or to technical issues so that no women were excluded at this stage of the process due to invalid test results. Pelvic examination with VIA/VILI for HPV positive was well‐accepted. Woman that needed a treatment were considered as eligible for treatment (thermal ablation or LEEP), no woman stopped her treatment because of pain or discomfort and all of them have received the therapy (Figure 1).

### 3.4 Key performance indicators (KPI)

Over a 7‐month period (a) the rate of women screened was 8.4% (840/10 000) (target 20% for 12 months), (b) the HPV positivity was 19.8% (expected range 18%‐25%), (c) the number of HPV‐positive women who accepted to participate and received VIA/VILI and D‐VIA/VILI testing was 100% (target > 90%) and (d) the number of VIA/VILI‐positive women who accepted treatment and received the complete therapy (target > 90%. Other KPI registered in the 3T process were (e) the VIA/VILI positivity rate, which reached 50.6% (range 45%‐55%), (f) the rate of patient having a 3T process conducted in a single visit, which was 79.8% (target > 80%), (g) follow‐up at one month which was 96.4% (target > 80%) and (h) at 6 months which was 70.6% (target > 80%) (Table 3). At 6 months, 58.3% were tested positive for HPV and 25% of them were VIA/VILI positive (patients included in the study who were able to benefit from their visit at 6 months).

**Table 3:** Keys Performance Indicators (KPI) to monitor screening and treatment

Performance Indicator	Target/range	Status	Results
**Screening rate:**	2000/year	840/7 months	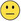
i. Target population Dschang ~ 10 000 women, 30‐49 years, in a five‐year period (screening coverage: 80%)	20%	8.4%	
Diagnostic and Treatment process			
ii. HPV positivity rate	18%‐25%	19.8%	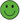
iii. Compliance to referral to VIA/VILI and complete test^a^	>90%	100%	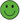
iv. Compliance to referral to thermal‐coagulation and complete treatment	>90%	100%	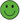
v. VIA/VILI positivity rate	45%‐55%	50.6%	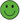
vi. Single visit from diagnostic to treatment	>80%	79.8%	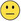
vii. Compliance to follow‐up at 1 month	>80%	96.4%	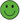
viii. Compliance to follow‐up at 6 months^b^	>80%	70.6%	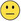

^a^ Once patient has been included in the study; considered as complete if only if the three sets of photos (native, acetic acid and Lugol) were performed and interpretable.

^b^ Patients included in the study who were able to benefit from their visit at 6 months.

## 4 DISCUSSION

We implemented a single‐visit 3T‐approach, based on Self‐HPV for primary screening followed by VIA/VILI triage and treatment with thermal ablation or LEEP if needed. This approach involving HPV test, VIA/VILI and treatment in a single visit needs women's compliance, as well as organizational factors such as adequacy of providers, scheduled service, functionality, and availability of material supply for completing the whole process. Public health interventions are complex especially in an approach using sequence of tests and treatments, therefore, in order to check the activity and determine if we achieved the desired objective, we introduced a limited number of indicators which are feasible in a primary and secondary level of care with the aim that it should cover the different steps from screening to treatment. It should also be easily understandable and measurable by providers and can be produced periodically.^9^, ^10^

Performance indicators for CC screening and treatment in LMIC are recommended by WHO and the Pan American Health Organization, but to date are still poorly reported in the literature.^9^, ^20^ The KPIs evaluated in our study consider different components (ie patient, provider and organizational factors) that should be reached in order to ensure that all steps from screening to treatment are performed and completed appropriately. They were focused on areas that are likely the most important in terms of improving patient outcomes and ensuring the most efficient delivery care.

We found that the rate of patients having a 3T process conducted in a single visit was 79.8% (target > 80%). Associating testing with an immediate offer of triaging and treatment for screen‐positive cases is probably the best way to reduce the loss of follow‐up.^5^ The compliance to VIA/VILI triaging and treatment of positive women was over 90% and might reflect extensive counseling and the opportunity to receive immediate treatment after VIA/VILI. Previous studies have demonstrated that loss of follow‐up between test and treatment is a real concern with loss ranging from 5% to 40% reducing program effectiveness.^21^, ^22^, ^23^ Our pilot analysis also demonstrates that the desired screening rate of the target population of Dschang could be achievable. To boost the participation rate, a selection and training of community health workers was planned to provide education on CC and its prevention in the community.

We found a VIA/VILI positivity rate of 50.6% among HPV‐positive women, which is higher to what was described in previous studies conducted in population with unknown HPV status,^24^, ^25^, ^26^, ^27^ ranging between 10% and 35%. This might be due to the IARC criteria considering only dense acetowhite lesion with sharp borders as positive.^3^ In our study, in order to maintain the high sensitivity obtained after HPV testing, we consider as positive any acetowhite lesion, localized in the transformation zone including also “faint” or “pale” lesions, which might explain the higher rate of positive cases.^12^ Future studies will be necessary to compare overtreatment rates between populations screened primarily by VIA and those screened by a 3T approach.

Our study has some limitations that need to be addressed. First, it was conducted in a single site with a relatively small sample size. Second, data need to be evaluated in a centralized office that serves different health facilities. Finally, it is a preliminary analysis, so results may not be generalized beyond the purpose of the study.

Strength of this study is the high quality of data collection as part of routine care, which allow to evaluate quality of the programs and formulate specific recommendations regarding the 3T‐approach.

Using KPIs measures is considered as having an important impact on patient safety, monitoring and quality improvement of a CC screening program.^10^ Although, the individual pathway may be different for every woman and achieving or missing a target does not necessarily suggest that women have received a poorer quality of care. Therefore, KPIs should be considered as variables that measure one aspect of the program and determine whether the targets or the standards have been achieved over a period of time. Moreover, KPIs for CC prevention in a LMIC context probably need constant review to ensure appropriateness and applicability.^10^

In conclusion, the performance of the 3T‐approach in rural Cameroon can be assessed with a minimum set of key indicators. The analysis demonstrated that the program works adequately whereas it is necessary to define a periodic assessment of KPIs to assure a continuous improvement. This study could help low income countries planning to scale up outcome indicators of interest for their program based on primary HPV testing and treatment in a single visit.

## CONFLICT OF INTEREST

None.

## AUTHOR CONTRIBUTIONS

MdP and JL led content development and writing of the article. PP and PV provided conceptual input, reviewed and edited article drafts. BK, EFT, and JTF wrote content on Cameroon, described the 3T procedure in Dschang and edited article drafts. RC provided statistical analysis and edited article drafts. JS and CF organized the health information system, provided data, and edited article drafts. The database is available as needed.

## Data Availability

The database on which the analysis of the study is based is available if required.
